# Awareness and readiness of health care workers in implementing Pradhan Mantri Jan Arogya Yojana in a tertiary care hospital at Rishikesh

**DOI:** 10.3126/nje.v10i2.27941

**Published:** 2020-06-30

**Authors:** Navuluri Kranthi Kumar Reddy, Yogesh Bahurupi, Surekha Kishore, Mahendra Singh, Pradeep Aggarwal, Bhavna Jain

**Affiliations:** 1 Junior Resident, Department of Community and Family Medicine, All India Institute of Medical Sciences, Rishikesh, Uttarakhand, India; 2 Assistant Professor, Department of Community and Family Medicine, All India Institute of Medical Sciences, Rishikesh, Uttarakhand, India; 3 Professor and Head, Department of Community and Family Medicine, All India Institute of Medical Sciences, Rishikesh, Uttarakhand, India; 4 Assistant Professor, Department of Community and Family Medicine, All India Institute of Medical Sciences, Rishikesh, Uttarakhand, India; 5 Associate Professor, Department of Community and Family Medicine, All India Institute of Medical Sciences, Rishikesh, Uttarakhand, India; 6 Senior Resident, Department of Community and Family Medicine, All India Institute of Medical Sciences, Rishikesh, Uttarakhand, India

**Keywords:** PMJAY, Ayushman Bharat, Awareness, Readiness

## Abstract

**Background:**

Right to health is one among the important components of basic human rights. The Government of India had announced “Ayushman Bharat for a new India-2022”, during 2018-19 parliament budget sessions with two components namely, Health and wellness centers for strengthening primary care and national health protection scheme now known as “Pradhan Mantri Jan Arogya Yojana (PMJAY)” for enabling access to secondary and tertiary healthcare services. Current study was conducted to assess awareness and readiness of the health care workers in implementation of Pradhan Mantri Jan Arogya Yojana

**Materials and Methods:**

A hospital based cross sectional study was conducted with estimated sample size calculated of 236, with treating consultants and residents as study participants. Participants filled a self-administered pretested semi-structured questionnaire to assess the level of awareness and readiness in implementing PMJAY. Data was entered and analyzed using EPI Info 7 software.

**Results:**

Total number of participants were 181. Mean awareness score was 4.5±1.96 and mean readiness score was 16±5. Mean awareness and readiness score among medical and surgical branches was not statistically significant. There was significantly high awareness score among faculty as compared to senior residents. Relationship between awareness and readiness was found to be correlated with Pearson’s correlation of 0.206 and was statistically significant. Linear regression model demonstrated an increase of 0.531 units in readiness for every unit increase in awareness score.

**Conclusion:**

Mean awareness score of doctors was just around half of maximum possible score. Awareness is more among faculty members than residents. With increase in awareness there is an increase in readiness among the study population. There is a need to organize workshops on PMJAY for stakeholders.

## Introduction

Right to health is one among the significant components of basic human rights. Everybody on the earth should be able to access preventive and curative aspects of health for a happy and productive living. World health organization (WHO) theme for the year 2019, “Universal Health Coverage (UHC) – Everyone and Everywhere”, envisages that access to healthcare services without any financial constraints [1]. UHC is also one of the key components of United Nations Sustainable Development Goals [2].

National health policy and various National Health Programs in India had a lot of success stories such as eradication of diseases like small pox, neonatal tetanus, controlling diseases like malaria, and reduction in maternal mortality and infant mortality. But these programs proved unsuccessful for provision of quality healthcare services to the community, particularly in rural communities [3]. Also, due to financial inequality there is huge gap in accessing the curative aspects of health in a developing country like India.

It was estimated that nearly 6% population of India, were unable to seek curative services due to financial reasons [4]. Even if they wish to seek health services, those people are experiencing catastrophic costs dragging them into further more poverty. A large proportion of Indian population i.e. 85% is not insured by any health insurance [4]. Also, spending on health in India is only 1% of the gross domestic product (GDP), which is lowest globally. As a result, India is facing short comings related to workforce, infrastructure and availability of quality care in Indian Health care system [5].

The Government of India had announced “Ayushman Bharat for a new India-2022”, in 2018-2019 Parliament budget sessions. It has two major initiatives, construction of “Health and Wellness Centers (HWC)” for strengthening primary care and National Health Protection Scheme now named as “Pradhan Mantri Jan Arogya Yojana (PMJAY)” for enabling access to secondary and tertiary health care services [6]. This scheme intends to offer financial protection to nearly 500 million vulnerable Indians and also prevents 50–60 million Indians to plunge into impoverishment due to escalating healthcare expenses. Uttarakhand is one of the states which are implementing Pradhan Mantri Jan Arogya Yojana (PMJAY) [7].

Success of this program depends on institution being adequately well equipped with resources in implementation, delivery, and monitoring of the scheme. Regular monitoring of Program is necessary so as to make sure it is getting implemented in a sustainable manner. Scrutiny of adequate infrastructure in institutions is required and also readiness among the health care providers is assessed who are crucial in effective implementation of the program. Hence to ensure adequate readiness there is a need to generate awareness among health care providers regarding administrative and programmatic aspects along with impact of PMJAY on Indian Health system.

In this context we planned a study with an objective to assess knowledge and readiness of health care providers regarding PMJAY in an empanelled tertiary care hospital at Rishikesh. This helps in assessing the level of awareness and readiness among the study participants and planning of further steps by the institution for hurdle free implementation of PMJAY.

## Methodology

### Study design and participants

A cross sectional study was conducted in AIIMS Rishikesh, which is one of the empanelled hospitals with PMJAY, with a mandate to provide tertiary care services. It has consultants and residents working 24/7 in almost all major specialties catering a large number of patients below poverty line and probable beneficiaries of PMJAY. For the present study we included consultants and resident doctors involved in patient care as study participants. Study was conducted for a period of 2 months from December 2018 to January 2019.

### Inclusion criteria

The study participants were faculty members, senior residents or junior residents working at AIIMS Rishikesh.

### Exclusion criteria

Participants who were not directly involved in patient care and denied to give consent for the study were excluded.

### Explanatory variables

Data on speciality and designation were collected. Specialities were categorized as Surgical and medical department. Surgical department included all broad and super specialties related to surgery. Medical department included all remaining departments.

### Outcome variable

Awareness Score: It is calculated from the responses given by the participant for the questions assessing knowledge of participant on PMJAY. A response of yes was given a score 1 and the response of no or don’t know was given a score 0 and sum of the scores was calculated as Awareness score of the participant. Readiness Score was calculated by adding responses for the items related to readiness in the Likert scale.

### Data collection:

Predesigned, pretested and semi-structured questionnaire was used for data collection. Written informed consent was obtained from study participants and confidentiality was maintained.

### Questionnaire design and validation:

Questionnaire constituted items on knowledge and readiness based on “Operational Guidelines on Ayushman Bharat National Health Protection Mission (AB-NHPM)” [9]. There were 9 items in the questionnaire to assess awareness (yes, no or don’t know format). Maximum possible score for Awareness was 9. Readiness was assessed using Likert scale with 5 components and maximum attainable score for readiness was 25. For validation, the study tool was reviewed by seven subject experts and they were requested to rate each item on 4-point rating scale with “4-highly relevant, 3-quite relevant, 2-somewhat relevant and 1-not relevant”. Content validity index was calculated for each item based on their rating. It was found that Mean I-CVI was 0.98 and Mean expert proportion was 0.98. With this mean I-CVI and mean expert opinion the study tool was validated. [10] Bases upon content validity questionnaire was modified.

#### Ethical committee approval:

Ethical approval was obtained from Institutional ethical committee with vide letter # 309/IEC/2018.

### Sample size calculation:

In absence of documented evidence, we assumed 50% proportion of study population will have adequate readiness score. By using Stat Cal module of Epi Info 7 for android with 95% confidence interval and absolute precision of 5%, we estimated that minimum required sample size of 176 for a study population of 325 [8].

### Data management and statistical analysis:

Epi Info 7.0 version for windows was used for data entry and analysis. Descriptive statistics was represented with means ± SD, median, proportions. Awareness score and readiness scores were compared using One-way ANOVA followed by post hoc test Bonferroni. Pearson’s correlation followed by linear regression was applied to assess relation between awareness and readiness scores. P-value less than 0.05 was considered statistically significant

## Results

Total number of participants were 181. Among the respondents 63% were junior resident doctors followed by 28% senior resident doctors and 9% faculty. 50.8% respondents belonged to medical specialities and 49.2% surgical specialties. All the participants had knowledge regarding PMJAY scheme. None of the participants received any training on PMJAY. The overall mean awareness score was 4.5±1.96 and mean readiness score was 16±5.

Table 1 shows the comparison of awareness scores and readiness scores according to the departments and designation of the participants. When the awareness scores were compared among various departments, the difference between medical and surgical branches was not statistically significant. When awareness score was compared among different participants, faculty had significantly more awareness as compared to senior residents. However, no statistical significance in readiness score was found when various departments and different groups of study population were compared.

The relation between awareness score and readiness score was found to be statistically significant and showed a positive correlation (r=0.206, p=0.005). Linear regression model was generated to quantify the amount of change in readiness score with change in awareness score. It was observed that there is an increase of 0.531 units in readiness for every unit increase in awareness score with a p value of 0.005(Table 2).

## Discussion

### Summary of findings

Awareness scores were better among faculty than residents, which might be due to less programmatic information. There is a statistically significant increase in readiness scores with increase in awareness scores. This shows that awareness can be much better, if training and awareness sessions were conducted prior to implementation of program.

### Implementation of similar schemes in past

Success of any program depends on availability of adequate resources and proper training of stake holders as well as their involvement right from the initiation of program to point of service delivery [11]. PMJAY was one of initiative of Ayushman Bharat which is deemed to be ambitious and valiant by Government of India and it would be tough for Indian health care system to implement it [12]. Previous experiences from similar initiatives in past proved to be ineffective due to suboptimal implementation and partial scale up [13].

Earlier health insurance schemes such as RSBY was also not successfully implemented this may be due to decreased commitment and lack of involvement at point of service delivery [14], which was again overlooked. So, it is important to ensure awareness and training of health care providers regarding the scheme, right before initiation of it which might have increased readiness of health care workers. This can be further improved by taking inputs from health care providers in understanding ground level constraints that might happen during implementation of scheme.

### Importance of PMJAY for Indian Healthcare System

Indian health care system faces a lot of challenges like, insufficient funding resources, non-availability of better infrastructure, lack of oversight of health care provision [5]. Private healthcare sector has also become dominating over public healthcare sector in providing health services in India [15,16], and thus became an important part in PMJAY. But there is evidence across similar health care systems of India that private health care providers might deviate from evidence-based practice to unnecessary testing and treatment practices [17,18]. When PMJAY is providing financial assistance, these unethical practices might increase which will create more burdens on government. Unethical practices at all levels right from point of delivery of services to high level program managers and decision makers remains an unresolved issue [19].

Public health managers should take steps to organize awareness programs in the form of CME or training programs for health care providers to fill the gaps in delivery of services under PMJAY. Involvement of all stakeholders to be ensured. Periodic review and assessment of program by stakeholders to be ensured. External auditing about program implementation should be adopted by all empanelled hospitals to ensure that tax payer’s money is not wasted.

## Limitation of the study:

1. Use of Convenient sampling method which led to improper distribution of study participants.
2. PMJAY was launched across all states in India, and implementation differs from one state to other. Our study was based on single center so results cannot be generalized.

## Conclusion

Even though there is an intense IEC regarding the benefits of the scheme, mean awareness score of the doctors was very low i.e. less than half of maximum possible score. Awareness was more among the faculty members than residents. There is no significant difference in readiness between residents and faculty members. With increase in awareness there is an increase in readiness among the study population.

## Figures and Tables

**Figure 1:**
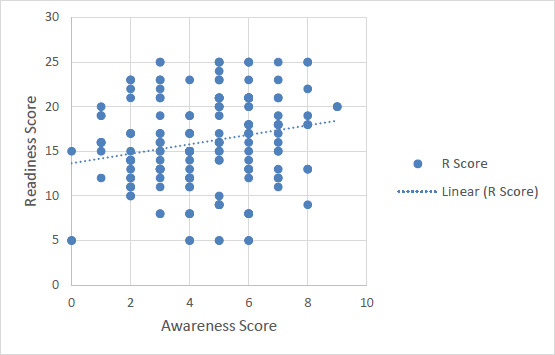
Relationship between readiness score and awareness score

**Table 1:** Comparison of awareness scores and readiness scores department wise and designation wise

**Awareness Score**	**Department**	**N**	**Mean ± SD**	**P value**
	Medical	92	4.5652 **±**2.17531	0.696
	Surgical	89	4.4494 **±**1.77105	
	Designation*			
	Junior resident	114	4.5526 **±**2.17830	0.04
	Senior resident	50	4.0800 **±**1.49612	
	Faculty	17	5.4706 **±**1.50489	
**Readiness scores**	**Department**			
	Medical	92	16.3478 **±**5.21923	0.427
	Surgical	89	15.7416 **±**5.00801	

*Oneway ANOVA followed by Bonferroni

**Table 2:** Linear regression between readiness score and awareness scores

Dependent variable	Unstandardized Coefficients		P value
	B	Standard Error	
**Awareness score**	0.531	0.189	0.005

## Future scope of the study:

More training and awareness sessions should be conducted in all empanelled hospitals so as to fill gaps in knowledge. Time to time knowledge should be assessed by taking small sessions. This might help in increasing readiness among the healthcare provider thereby helping in achieving proposed goals of the scheme. Health care providers should be asked to give their opinions in improving this knowledge so that we can achieve desired goals

## What is already known on this topic?

PMJAY was a recent initiative by the Government of India to attain the goals of “universal health coverage (UHC)” and reduce the out of pocket expenditure for vulnerable population groups. Limited literature available on this topic.

## What this study adds:

This study gives an insight on awareness and readiness of health care workers regarding PMJAY. This information will help policy makers and health managers to take necessary steps for better implementation of scheme.
